# Acceptance and barriers pertaining to a general practice decision support system for multiple clinical conditions: A mixed methods evaluation

**DOI:** 10.1371/journal.pone.0193187

**Published:** 2018-04-19

**Authors:** Derk L. Arts, Stephanie K. Medlock, Henk C. P. M. van Weert, Jeremy C. Wyatt, Ameen Abu-Hanna

**Affiliations:** 1 Academic Medical Centre, Department of General Practice, Amsterdam, The Netherlands; 2 Academic Medical Centre, Department of Medical Informatics, Amsterdam, The Netherlands; 3 University of Southampton, Wessex Institute for Health Research, Southampton, United Kingdom; University of Stirling, UNITED KINGDOM

## Abstract

**Background:**

Many studies have investigated the use of clinical decision support systems as a means to improve care, but have thus far failed to show significant effects on patient-related outcomes. We developed a clinical decision support system that attempted to address issues that were identified in these studies. The system was implemented in Dutch general practice and was designed to be both unobtrusive and to respond in real time. Despite our efforts, usage of the system was low. In the current study we perform a mixed methods evaluation to identify remediable barriers which led to disappointing usage rates for our system.

**Methods:**

A mixed methods evaluation employing an online questionnaire and focus group. The focus group was organized to clarify free text comments and receive more detailed feedback from general practitioners. Topics consisted of items based on results from the survey and additional open questions.

**Results:**

The response rate for the questionnaire was 94%. Results from the questionnaire and focus group can be summarized as follows: The system was perceived as interruptive, despite its design. Participants felt that there were too many recommendations and that the relevance of the recommendations varied. Demographic based recommendations (e.g. age) were often irrelevant, while specific risk-based recommendations (e.g. diagnosis) were more relevant. The other main barrier to use was lack of time during the patient visit.

**Conclusion:**

These results are likely to be useful to other researchers who are attempting to address the problems of interruption and alert fatigue in decision support.

## Introduction

Clinical decision support systems (CDSS) are computerized tools that assist in making clinical decisions [1]. In recent years, many studies have investigated the use of CDSS as a means to improve care [2–5]. The interest in CDSS as a strategy to implement guidelines is not surprising given the ever-increasing number of clinical practice guidelines. For example, the Dutch College of General Practitioners (NHG) currently offers more than 100 guidelines to its members. It is not unlikely that, for a given patient, more than 15 guidelines might apply. It is this complexity that creates the need for CDSS, especially in older patients with many comorbidities where guidelines often overlap or even contradict each other [6].

Despite the apparent advantages of computer-based CDSSs, most systems have thus far failed to show significant effects on patient-related outcomes [2–4]. A recent review did however find moderate improvements in morbidity outcomes [5]. Reviews that studied CDSSs in long term conditions such as asthma, diabetes and hypertension did not show an impact on patient outcomes [7–9]. Therefore evidence for the cost-effectiveness of CDSSs is lacking [2]. A limitation reported in all reviews investigating the effects of CDSSs is the fact that studies are often of poor methodological quality. Another limitation is that many studies focus on systems that provide support for a single disease, while future systems will be required to support multiple guidelines and the care of patients with multiple diseases.

Many reasons for the low usage and/or effectiveness of CDSSs have been identified. These include: lack of usability, lack of integration with host systems, lack of time to effectuate advice, inapplicability to the patient, lack of integration with current workflow, and alert fatigue [10, 11]. These reasons partly explain the limited effectiveness of CDSSs, but often we do not know why apparently high-quality systems go unused or are ineffective [12].

We attempted to address many of these issues in a CDSS that was developed for the current project. This system was implemented in general practice and was designed to be both unobtrusive and to respond in real time to the user’s actions in the record system, drawing on up to 15 validated clinical decision rules [13]. For instance, when a general practitioner (GP) added a diagnosis of atrial fibrillation, the system would be triggered and evaluate antithrombotic treatment for stroke prevention. Thus the GP would receive immediate feedback and could respond by modifying the prescription, unlike most systems where the user receives feedback only after the order is completed and must backtrack to follow the advice. Our hypothesis was that this pre-emptive mode of operation would increase user adherence to system advice, as the GP would not have to modify already prescribed medication.

Despite the features mentioned above, which attempted to address the problems reported in many CDSS studies [10, 11], we found low overall usage, which declined over time: The CDSS generated an average of 15 notifications per working day for each GP in the field test. However, GPs only clicked on a total of 4119 of the 126158 notifications, a click rate of 3%. The effectiveness of the system was limited and differed greatly between decision rules. Statistical evaluation of the usage and effectiveness of the system will be reported elsewhere; however, that analysis gives no insights into the reasons for the GPs’ limited usage of the system, nor how to improve it in future. Thus, the objective of the current study was to perform a qualitative investigation as a follow up to our CDSS clinical trial, using a survey and focus group evaluation, with the primary goal of identifying remediable barriers which led to disappointing usage rates for our system.

## Material and methods

### Clinical decision support system

The trial protocol for our randomized controlled trial is described elsewhere [14, 15]. Briefly, the system included two guideline domains, one relating to care of older adults [13] and the other to anticoagulant management in atrial fibrillation [16]. All these guidelines were implemented as clinical decision rules in a single rule-based system and coupled with coded data in the GP’s electronic patient record system. The rules to be included in the system were based on a Dutch version of ACOVE (Assessing Care of Vulnerable Elders) rules and selected with input from target users by way of a survey. The final rule set consisted of 30 decision rules relating to the two domains covering diagnoses such as atrial fibrillation (AF), diabetes, hypertension and medication prescriptions.

**Fig 1:**
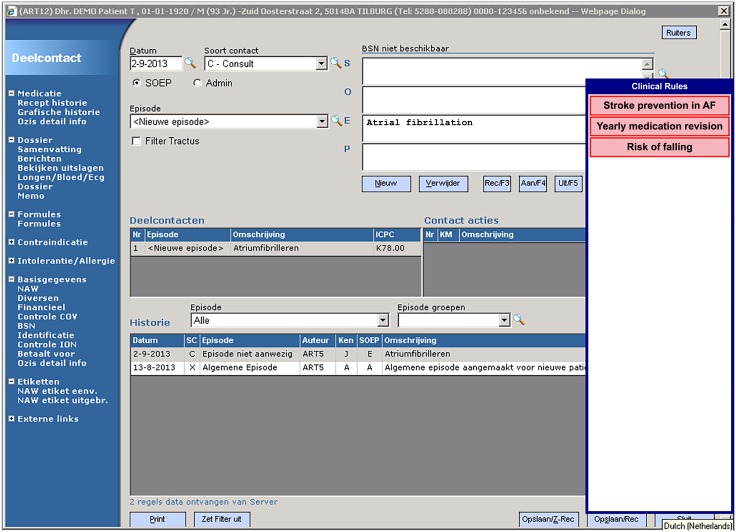
The notification window in its expanded state, showing three notification items.

**Fig 2:**
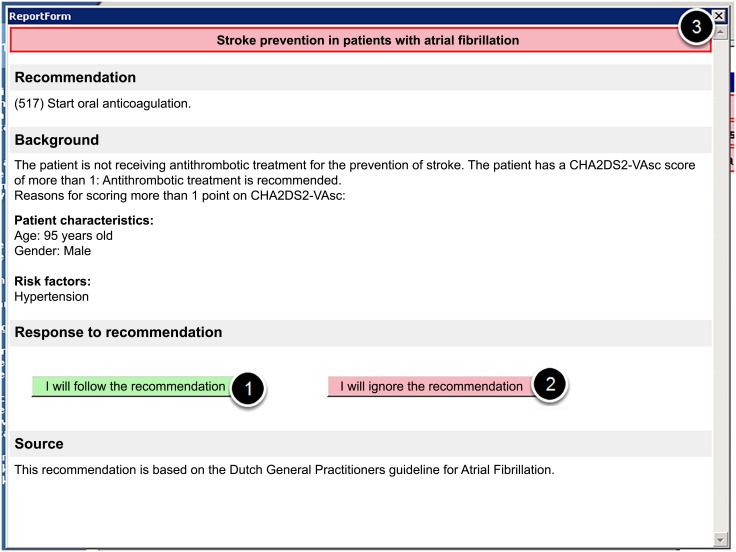
The window after clicking on a notification item, containing background information, an actionable recommendation and response buttons to allow the GP to indicate whether they accept (1) or decline (2) the advice (3) close the window (no action).

User notifications about CDSS recommendations were shown in a floating window, that could contain up to 15 items. Each item contained a short (1 to 3 word) description of the corresponding recommendation (Fig 1). It was not uncommon for there to be more than 5 recommendations for an elderly patient (median: 4). The notification window was collapsed by default, and in this state only two letters of each notification item could be seen. The window could be expanded temporarily by moving the mouse cursor over the window (mouse over). The user could drag the window to either the left or the right side of the screen. On clicking a notification item, a window appeared containing information about the recommendation: background information, the recommendation itself, and buttons to allow the GP to either accept or ignore the advice (Fig 2). All activity in the system was logged, this included mouse movements, opened recommendation windows and responses to recommendations.

### Data collection

**Fig 3:**
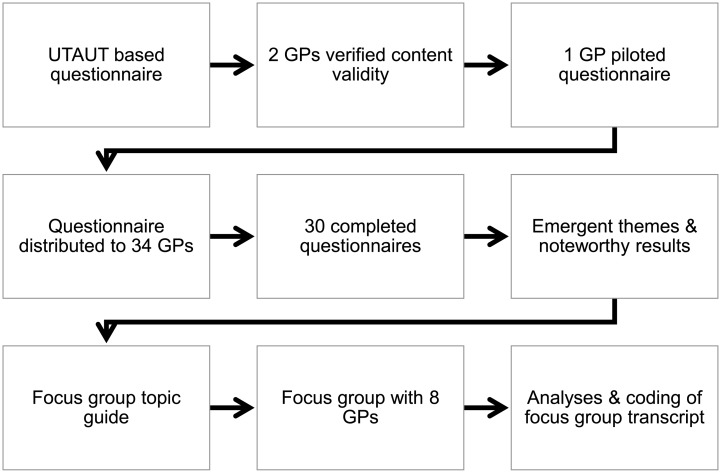
Overview of activities related to data collection.

Fig 3 shows an overview of the activities related to the development of the questionnaire and focus group topic guides.

### Questionnaire

A questionnaire was developed to investigate user attitudes about the CDSS. Of the 40 survey questions, 28 represented operationalisations of concepts from the unified theory of acceptance and use of technology (UTAUT) [17]. The question phrasing was based on known barriers and facilitators for CDSS use [10, 11]. An additional 6 questions were added to explore new areas of interest identified in the pre-implementation survey [18], and 6 more were added to explore user views on specific system features, such as the real-time feedback. A validation focus group with two GPs was performed to review content validity which was then piloted on one GP. The questionnaire contained three free text questions.

### Questionnaire distribution

The online questionnaire was distributed by email to all GPs enrolled in the trial. To maximise response rates, GPs received a small reward (~12 euros) for completing the questionnaire, and an additional similar reward if an overall response rate of 85% was reached. E-mail reminders were sent every two weeks for a maximum of four times, after which the remaining non-responders were approached by phone to remind them about our questionnaire. Participants were required to enter their name so we could associate their age and gender with the survey results using demographics data from the GP centres.

### Questionnaire analyses

Two authors (DA, SM) independently reviewed the survey free text comments and extracted themes, representing clusters of topics, by induction [19]. Quantitative survey results were also independently reviewed by two researchers, who selected results from the survey which might be clarified by discussing them in the focus group that followed. These selections were compared and merged during a consensus meeting between DA and SM, and used as input for the focus group.

### Focus group

To further clarify the free text comments and receive more detailed feedback from GPs, a focus group was organized. We created a topic guide structured according to the Information Systems Success Model by DeLone and McLean, which consists of six dimensions of information systems success [20]. The topic guide consisted of items based on results from the survey and additional open questions aimed at encouraging free discussion about the system. The 8 invited GPs were sampled purposively, based on their answers to the survey. All GPs had used the system more than once, but we specifically chose GPs with either a lot or a little self-reported experience using the system. Furthermore, we prioritised GPs as focus group participants if they had given more detailed feedback on the free text survey questions. The focus group started shortly after normal clinic hours, and food and drinks were provided. The focus group took 90 minutes, and was recorded using video and audio.

Recordings were transcribed verbatim, and the transcriptions coded independently by two researchers using the previously-identified topics as an initial coding scheme (from the Information Systems Success model, topics from the survey, and known issues with the system) and coded using MaxQDA 12 [21]. Comments that were relevant to the central questions (why the GPs did or did not use the system, and suggestions for improvement) but did not fit into any of the previously identified categories were also noted, then classified into emergent themes.

### Ethical approval

We did not seek ethical approval as this study did not involve any patients.

## Results

### Questionnaire

#### Respondents and response rates

The response rate was 94% with 32 responses out of 34 eligible respondents. Four additional respondents were invited but were unable to respond due to switching jobs, retirement or maternity leave. Two surveys were not completed fully, resulting in a total of 30 surveys that were included in the analyses. Respondents were on average 51 years old (SD: 9.5 years) and 63% were female.

#### Self-reported usage

**Fig 4:**
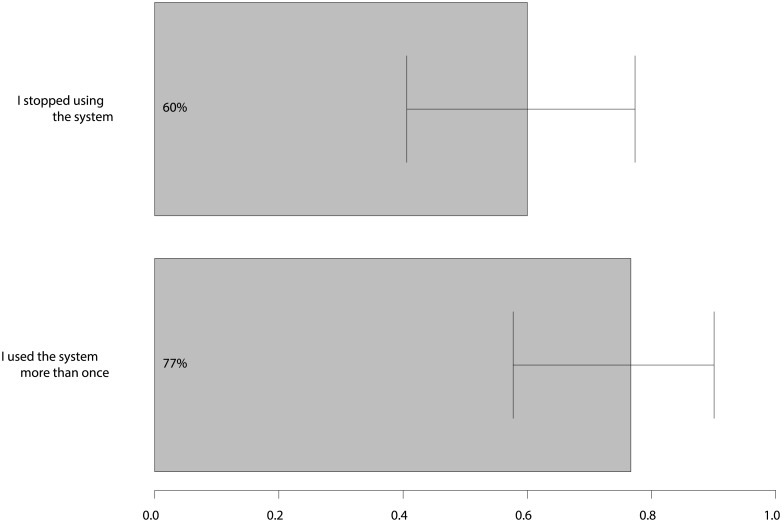
Self-reported use of the system. Percentage of users that answered yes to the listed questions.

**Fig 5:**
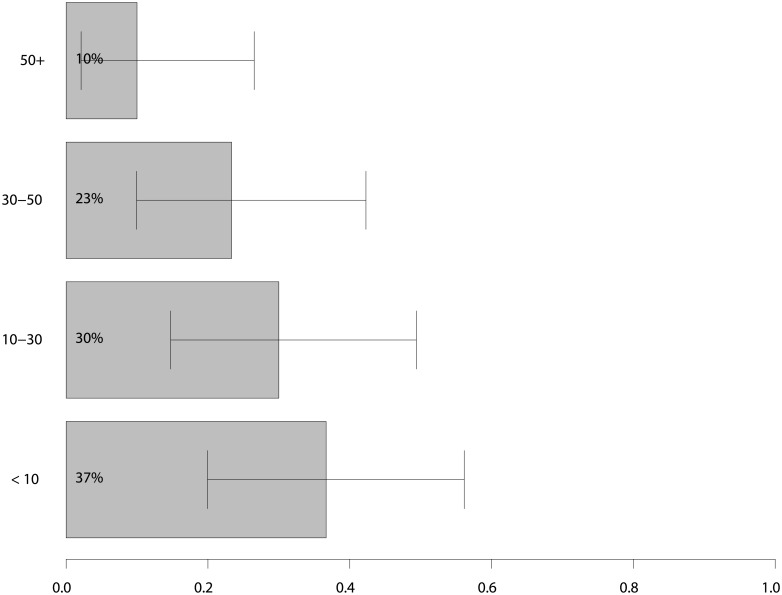
Self-reported usage rates of the system. Answers to the question: “How often did you use the system, e.g. clicked a notification, accepted a recommendation etc.?”.

Most participants (23, 77%) used the system more than once, but 18 (60%) stopped using the system during the trial. Only 3 (10%) users reported having had more than 50 interactions with the system. Figs 4 and 5 contain additional statistics about self-reported usage of the system. We excluded survey results from 7 participants who reported not using the system more than once from further analyses, leaving 23 out of 30 surveys to be analysed. The reason for this exclusion was that these users gave neutral answers to our questions and did not answer open questions.

#### Use of instruction materials

The participants indicated that 70% had read the manual, 74% attended the live demo, and 39% watched the instruction video. Table 1 shows what combination of documentation was used by each participant

**Table 1:** Usage of various documentation formats.

Documentation used	N	%
Only PDF Manual	4	9%
Only Video	0	0%
Only Presentation	6	13%
PDF Manual & Video	2	4%
Video & Presentation	1	2%
PDF Manual & Presentation	4	9%
All documentation	6	13%

#### Quantitative survey results

**Fig 6:**
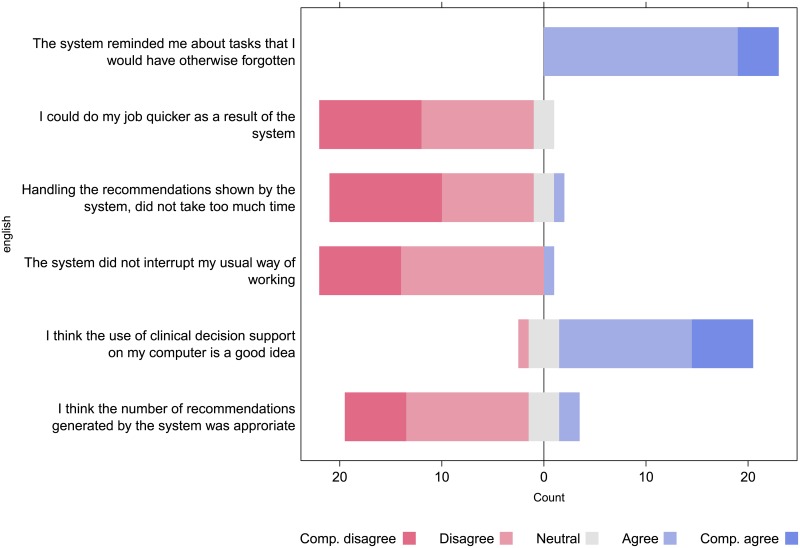
Selected questions and Likert scales.

The two researchers independently selected the same four survey results as possible input for further discussion and each selected one additional result. Upon discussion it was decided to include both of these results. The selected results are listed in Fig 6.

#### Survey free text comments

There were four free text fields in the survey. Both researchers independently identified the same emergent themes in these comments.

The first theme that emerged in response to the question “What was the main advantage of using the system?” was that the system reminded and triggered GPs about things they would have or had forgotten. E.g. “Being notified of medical issues that sometimes fade into the background”. A second theme was the way the system encouraged the GP to systematically analyse the patient, e.g. “More—and more structural—attention for several issues that arise often in the elderly”.

Answers to the second free text question “What change would you want to make to the system” often related to the fact that the recommendation window floated *always on top*, i.e. would float over every other window the GP was using. Another often-made remark pertained to the long list of notifications for some patients, and the fact that GPs had no control over which notifications were shown. Other remarks included “Some recommendations overlapped with current protocols for diabetes and hypertension” and “The recommendations did not relate to a patient’s reason for visiting”.

The third free text question, “What were the most important reasons for not opening the recommendations?” was answered by more than 75% of GPs with an answer related to “Lack of time”. The second most common answer was “Too many alerts”. Two responses that were mentioned less often were “Recommendation not related to the patient or presented at the wrong time” and “Recommendation was not useful”.

### Focus group

Both coders identified the same topics as frequently-recurring. Each dimension of the Information Systems Success model is listed below with related topics and quotes for each topic to illustrate responses. One emergent theme was identified: “Requested features.”

#### System quality

The most frequently mentioned dimension (36% of coded phrases) was System Quality, particularly the quality of the interface (Table 2). The primary issue was that the floating window was always on top and thus blocked access to other applications. This frustrated many participants. The expanding notification window was experienced by some users as a “popup”. This is interesting as it only expanded on request (mouse over), indicating that any interruption on screen can register as a “popup”, i.e. something interrupting regular computer usage. The use of 2 letter abbreviations in the collapsed state wasn’t understood by all participants. Participants were divided on the utility of asking the user to document a reason when declining a recommendation. Some felt that the documentation was helpful in communicating their reasoning with other physicians in their practice and that feedback could be used to improve the guidelines; others felt it was “defensive medicine” and did not contribute to patient care. Participants also noted that it was clear that the CDSS was not fully integrated into the patient record system (e.g. medication could not be prescribed directly from the CDSS) and that this adversely affected the utility of the CDSS. Almost all participants felt that better integration with their EHR was essential, as they now felt they had to do the work twice: once in the CDSS, and then again in their own EHR.

**Table 2:** 

Topic	Quote
Always on top	“That was my biggest frustration, the [floating] bar on the left side.”
Always on top	“…also because the thing ([CDSS]) kept getting in the way, just it being in the screen was interruptive…”
Integration with host system	“‥ it has been said before but it would be great if the EHR remembers our actions [e.g. prescribing medication / ordering a test]. So that when you spend time thinking about your decision, that decision actually ends up in our EHR.”

Regarding our hypothesis about improving system usability, we note that (1) real-time feedback did not prompt specific discussion in the focus group, and (2) the interface design that we considered unobtrusive was still experienced by users as interruptive. To elaborate on the first point; the CDSS was designed to operate in real-time, i.e. using (on-screen) data that had been newly added to the patient file by the GP. This feature simply went unnoticed by the users, i.e. GPs did not notice that a new notification item appeared whenever they changed something on screen. Second, the system was designed to be non-interruptive by presenting a collapsed notification window on the side of the screen and requiring a user to actively open a recommendation. However, none of the participants felt that the system fulfilled that promise, and the system was experienced as interruptive despite our efforts to the contrary.

#### Information quality

Information quality was the second-most mentioned dimension, with the relevance of the recommendations as the most important topic (Table 3). Many GPs felt that recommendations that weren’t based on a specific diagnosis, e.g. only based on age or gender, were often irrelevant and took up too much time to be worthwhile. For example, recommendations that triggered on age alone were perceived as less valuable than those limited to patients with a particular diagnosis. These generic recommendations were usually preventative in nature. Participants also noted that many of the simple recommendations overlapped with protocolized and standardized care that was already being provided by physician assistants, such as reminders relating to diabetes foot care. Therefore, these recommendations did not improve care.

**Table 3:** 

Topic	Quote
Relevance	“There should be a distinction between alerts that are based on a diagnosis and alerts that are not”
Relevance	“…or just [triggering] on age, when you have a healthy active older person in front of you, that is completely useless.”
Relevance	“…we should look at what care is already being offered by local nurses and physician assistants; I think many of these [simpler] alerts should be removed because we are already aware of those patients.”

#### Service quality

Service quality was rarely mentioned (Table 4). Participants seemed satisfied with both the documentation provided and the availability of support, although they indicated that the user manual was too long (22 pages including many images).

**Table 4:** 

Topic	Quote
Documentation	“Now, I must say that I found the instruction film very clear… and it was also fairly short, not too long, it was very nice.”

#### Intended use

The dimension “Intended Use” included barriers and facilitators to using the system and following the system’s advice (Table 5). The most important topic for this dimension was a lack of time during the appointment to perform the suggested actions. Some participants indicated that they had scheduled follow-up appointments to address the additional recommendations. The number of recommendations was also mentioned as a factor, although participants did not agree on whether the number of recommendations should be limited as long as all recommendations were relevant.

**Table 5:** 

Topic	Quote
Time	“Due to simple time pressure I often didn’t use it, and dragged the window away to a point where I no longer saw it.”
Time	“But that plays into a different problem… We have now, and function now, with increasingly complex patients, and higher demand on care, more responsibilities too… and we still cram this into 10 minutes.”
Follow-up appointments	“I thought that was good, the [feeling of] ‘Oh yeah, that’s a lot, that’s really polypharmacy, I should do something about that.’ I used it as an alert and then asked people to come back to further explore the issue.”

#### Net benefit

Participants felt that the system did offer some benefit to themselves and their patients (10% of coded phrases) (Table 6). Participants felt that it reminded them to perform tasks such as medication review, or inclined them to review the patient record more thoroughly.

**Table 6:** 

Topic	Quote
Better/worse care	“I liked being obliged to do it—[to know] where precisely you stand, is there something wrong—I have everything in view. Done! But then you do want it to work flawlessly.”
Better/worse care	“Now, I have the feeling that it did have some benefit. That I carefully reviewed the whole patient, otherwise I wouldn’t have done that.”

#### Requested features

Participants mentioned several features that they felt would improve the system (Table 7). Participants wanted positive feedback when they had completed a task recommended by the system. The system would simply remove the recommendation from the list when the task was completed, but participants said they would instead prefer if the recommendation changed colour or gave some other indication that the task had been done correctly. Another required feature is the ability to add reminders to a “to do” list, so that they could be dealt with outside of the time constraints of the patient visit. Finally, clinicians would like to be able to customize the recommendations: removing recommendations that they did not think were useful, and ideally adding their own recommendations to the list.

**Table 7:** 

Topic	Quote
Positive feedback	“It would be nice if we would get a small confirmation that we’re doing the right thing, like a green notification or something?”

Table 8 contains a summary of our findings and a justification.

**Table 8:** Summary of findings and justification.

Recommendation	Justification
Thoroughly test usability in final setting.	User experienced the "always on top" nature of the system to be highly annoying. This could have been prevented by more thoroughly testing the system with end users in the final setting.
Integrate, as tightly as possible, with the host system, while avoiding overlap with existing alerts.	Users were disappointed by the fact that accepting a recommendation did not result in the recommendation being effectuated in their system. They saw this as an opportunity to reduce administrative load. Enabling this behaviour can increase user acceptance. Of note, studies have shown that CDSS integrated in EHRs are less effective, this is likely due to existing alerts in the EHR, thus overlap should be avoided.
Implement alerts based on specific criteria.	Our users strongly agreed that alerts based on demographics alone were significantly less useful than those based on diagnoses, medication or lab data. To reduce alert fatigue and increase user acceptance, alert triggering criteria should be as specific as possible.
Allow user prioritization or alert selection.	Users indicated that simultaneously presenting alerts for different disease areas was not effective for them. They reported that they would rather switch from alert to alert for 1 month at a time, and focus on one area.
Allow for different modes of presentation.	Users indicated that they would often prefer to be able to go over the entire list of alerts that was shown during the day, rather than only being presented with a list when the patient file was open. They would have preferred to use some quiet time to go over all patient alerts and select the ones that they felt were of importance.
Provide high quality training material through different channels.	Our users appreciated the different training opportunities (face to face, online video, PDF). It allowed all users to be informed about the system in a way they preferred. Other studies showed that lack of knowledge about the CDSS can limit effectiveness, thus emphasizing on training is an easy way to increase user acceptance.
Allow for positive feedback in the system.	Although of lesser importance than the other points mentioned, users indicated that they would also appreciate getting positive feedback to offset the alerts indicating they were "doing something wrong". Not all users agreed on this point, some indicated that providing high quality care was enough reward in itself. We recommend therefore that this option is made configurable.

## Discussion

This study used both qualitative and quantitative methods to identify barriers for usage of a primary care clinical decision support system. Despite strenuous efforts to create a non-interruptive CDSS, the system was nonetheless perceived as interruptive. Although the window displaying the recommendations was generally minimized to a thin bar, users did not like that it was “always on top”. They were divided on the utility of recording a reason for declining a recommendation, which was required for some recommendations. They noticed that the CDSS was not fully integrated into their EHR, leading to the need to do work twice (first accept the recommendation in the CDSS and then perform the action in the EHR). Participants felt that there were too many recommendations and that the relevance of the recommendations varied. They specifically noted that preventative care recommendations based only on age tended to be irrelevant, while risk-based recommendations based on more specific criteria such as a diagnosis were more relevant. The other main barrier to use, although not directly related to the CDSS, was lack of time to perform the suggested actions during the patient visit. One user handled this by scheduling additional visits. Despite these problems, users felt the system did make some improvement to care, particularly by reminding users of tasks that would otherwise be forgotten and in encouraging more thorough assessment of older patients. Users remained positive about the utility of CDSS in their practice. Participants felt that the system would be improved by adding specific, visible feedback for tasks performed correctly, allowing recommendations to be moved to a task list that could be handled outside of the patient visit, and facilities for customizing which recommendations appeared.

### Strengths and limitations

Two researchers performed all qualitative analyses and results were compared, reducing the risk of bias in this process. Use of established frameworks for understanding system usage (UTAUT and IS Success model [17, 20]) allows structuring of the results, while additional use of emergent categories ensures that no important topics are missed. Using a survey allowed us to efficiently gather feedback from all users, and following this with a focus group allowed us to gather a more nuanced understanding of the users’ experience and views.

However, this study does have some limitations. First, the survey instrument was not validated, although it was based on the validated UTAUT model of system use and content validity was established by two GPs. Our sample size was fairly small (37 GPs in the participating practices) and limited to a subset of the GPs in the country, but survey response rate was very high (all but two GPs completed the survey, and all participants invited to the focus group attended). We selected participants for the focus group based on their responses to the survey rather than randomly. This likely excluded users who were less interested in giving feedback about the system, but this was a conscious decision in order to maximize the utility of the focus group.

### Comparison to other studies

Many studies on CDSS failed to show user uptake and effectiveness in daily practice. ‘NHGDoc’, an EHR integrated CDSS co-developed by the Dutch College of General Practitioners, was implemented in over 65% of Dutch GP practices for various systems but had a usage rate of only 0.24% [22]. A focus group revealed the main barrier in that study was lack of awareness of the CDSS and its capabilities. Their large scale implementation likely made it harder to properly train participants, something that wasn’t an issue in our current study. Barriers that did play a part in usage of both CDSS related to high intensity of recommendations and the number of recommendations perceived as irrelevant. Other barriers that were mentioned by GPs in both studies included lack of customizability and the system not functioning optimally, leading to participants quickly giving up on the system. Although not explicitly mentioned by our participants, alert fatigue [23] might have played an important role in the lack of usage we found. GPs received a median of 4 notifications per patient, and although unsolicited popups were not used, the notifications were visible at all times. A recent trial by Cook et al. [24] also suspected alert fatigue acted as a barrier for usage, as did Lugtenberg et al. [22, 24]. However, popups can be more effective in reducing error, despite often being perceived as annoying. The challenge lies in identifying what recommendations warrant popups.

Another possible factor for lack of effectiveness mentioned by recent reviews is that CDSSs integrated in an EHR were less effective than stand-alone CDSS implementations, in both CDSS related to drug prescribing and in general [25]. One explanation for this finding is that EHR systems tend to have many alerts already. Users of these systems may already be experiencing alert fatigue, and new alerts may be more likely to be ignored than alerts introduced as part of an entirely new system [10]. The relative ease of integrating CDSS into an EHR may also lead to a lower threshold for adding an alert into the system, leading to the inclusion of less relevant alerts, which in turn aggravates alert fatigue [26]. An additional reason could be that an integrated system uses EHR data to provide recommendations and these data may be incomplete or inaccurate [27]. Therefore, lack of effectiveness may also be related to repeated erroneous recommendations [28]. To put it simply, without high quality data, CDSS cannot provide accurate recommendations.

### Interpretation, implications, impact

These results are likely to be useful to other CDSS researchers and system designers who are attempting to address the problems of interruption and alert fatigue in decision support [29, 30]. Although our system was carefully designed to be non-interruptive, it was not perceived as such by the users. The “always on top” feature contributed to this impression, as well as expanding on mouse over, which was perceived as a popup and hence "interruptive". An additional prototyping stage may have revealed these problems earlier and allowed consideration of different design choices. Users were also aware of the incomplete integration with the electronic patient record system, particularly when the CDSS asked them to document care but that documentation was not carried over to the EHR. Despite the fact that integrating CDSS in existing EHRs might reduce effectiveness, we are confident that tight EHR integration is the way forward. Tight integration means only one system providing recommendations and the ability to perform actions directly from a recommendation (e.g. ordering a blood test).

Perhaps most interesting is that the users differentiated general recommendations which triggered on age alone (e.g. ‘vitamin D prescription for elderly patients’) from recommendations relying on more specific criteria, such as a diagnosis. The latter were perceived as much more relevant. There seemed to be some overlap between whether the GPs felt they had enough time to handle the recommendations, whether there were “too many” recommendations, and whether the recommendations were perceived to be relevant. Participants were willing to make time to handle more recommendations if they were all considered highly relevant to patient care. Thus, stricter selection of recommendations and prioritizing recommendations may increase the perceived usefulness of the system. Despite the problems with the system, users did see some value in it and felt it contributed to patient care. Further, they felt decision support was a good idea and indicated that they would be willing to try an improved version of the system.

## Conclusion

Decision support systems can meet an important need in future healthcare, with its many guidelines and high administrative load, and participants in our study acknowledged the potential these systems hold for healthcare. However, implementing these systems in daily practice for multiple domains remains challenging. Prioritization, user customization, tight EHR integration and strict selection of recommendations might improve CDSS effectiveness. The lack of time to handle recommendations during the patient encounter may be partly addressed by allowing users to move recommendations to a task list or through other modes of presentation (e.g. e-mail). More focussed research on features of multi-domain decision support systems is required to guide vendors towards effective real world implementations.
